# Supplementary material for: Drosophila miR-87 promotes dendrite regeneration by targeting the transcriptional repressor Tramtrack69
Source: PLoS Genet. 2020 Aug 7;16(8):e1008942. doi: 10.1371/journal.pgen.1008942 (PMC7439810; doi:10.1371/journal.pgen.1008942)
Supplement: S1 Fig — Dendrite regeneration in wild-type control (WT) and miR-87 knockout (miR-87) C4da neurons at the indicated time points. Scale bar = 100 μm. Genotypes: WT, ppk-GFP; miR-87, ppk-GFP; miR-87KO/miR-87KO. (PDF) [file pgen.1008942.s001.pdf]

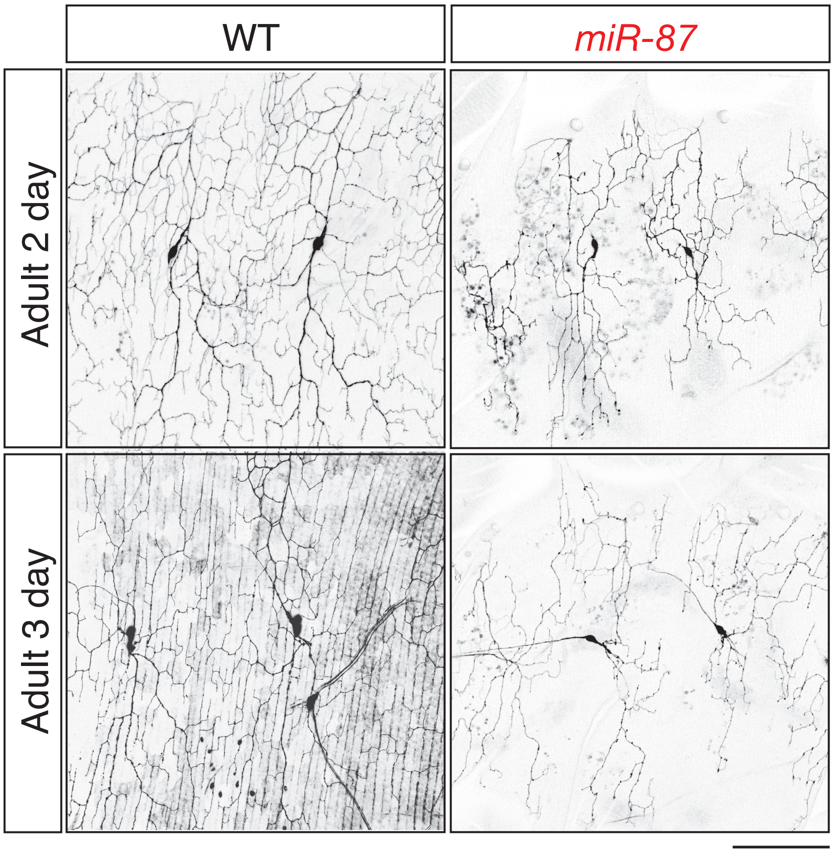

Supplemental Figure S1: *miR-87* is required for dendrite regeneration

Dendrite regeneration in wild-type control (WT) and *miR-87* knockout (*miR-87*) C4da neurons at the indicated time points. Scale bar = 100 μm.

Kitatani et al. Supplemental Figure S1
