# Supplementary material for: Drosophila miR-87 promotes dendrite regeneration by targeting the transcriptional repressor Tramtrack69
Source: PLoS Genet. 2020 Aug 7;16(8):e1008942. doi: 10.1371/journal.pgen.1008942 (PMC7439810; doi:10.1371/journal.pgen.1008942)
Supplement: S2 Fig — Total dendrite length of wild-type control (WT) and miR-87 overexpressing (miR-87 o/e) C4da neurons at white pupa (WP), 12 hours after pupal formation (12 hr APF), and 24 hours after pupal formation (24 hr APF). Note that no obvious difference was observed in dendrite pruning processes between WT and miR-87o/e neurons. Error bars indicate mean ± S.D; n = 15; n.s., not significant (unpaired t-test). Genotypes: WT, ppk-Gal4, UAS-mCD8GFP; miR-87o/e, ppk-GAL4, UAS-mCD8GFP;+/+; UAS-miR-87/+. (PDF) [file pgen.1008942.s002.pdf]

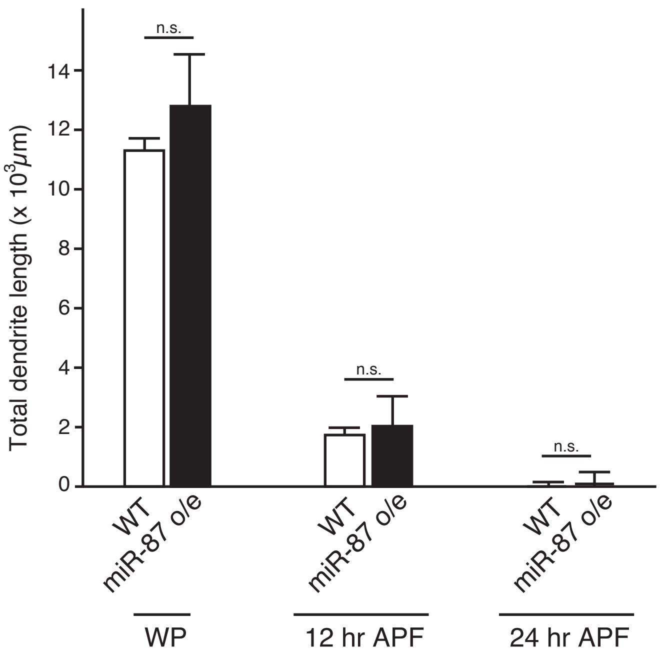

Supplemental Figure S2. Total dendrite length during pruning

Total dendrite lenght of wild-type control (WT) and *miR-87* overexpressing (*miR-87* o/e) C4da neurons at white pupa (WP), 12 hours afer pupal formation (12 hr APF), and 24 hours after pupal formation (24 hr APF). Note that no obvious defference was observed in dendrite pruning processes between WT and *miR-87* o/e neurons. Error bars indicate mean ± S.D; n = 15; n.s., not significant (unpaired *t*-test).
