# Supplementary material for: Drosophila miR-87 promotes dendrite regeneration by targeting the transcriptional repressor Tramtrack69
Source: PLoS Genet. 2020 Aug 7;16(8):e1008942. doi: 10.1371/journal.pgen.1008942 (PMC7439810; doi:10.1371/journal.pgen.1008942)
Supplement: S3 Fig — (A) Overexpression of hnf4 or br causes dendrite regeneration defects. Morphology of hnf4 overexpressing (hnf4 o/e) and br overexpressing (br o/e) dendrites at 72 hr APF. Scale bar = 100 μm. (B) Reduction of hnf4 or br dosage causes no obvious rescue in dendrite regeneration defects in miR-87 KO C4da neurons. Morphology of miR-87 KO with reduction of one copy of hnf4 (miR-87, hnf4/ miR-87) or br (br/+; miR-87/ miR-87) dendrites at 96 hrs APF. Scale bar = 100 μm. (C) Quantification of total dendrite length in control wild-type (WT), miR-87 KO (miR-87), miR-87 KO with reduction of one copy of ttk69 (miR-87; ttk69/+), miR-87 KO with reduction of one copy of hnf4 (miR-87, hnf4/miR-87), miR-87 KO with reduction of one copy of br (br/+; miR-87) dendrites at 96 hrs APF. n = 15, Error bars indicate mean ± S.D., *p<0.01 (ANOVA with a post hoc Bonferroni correction). Genotypes: WT, ppk-Gal4, UAS-mCD8GFP/+; miR-87, ppk-Gal4, UAS-mCD8GFP/+; miR-87KO/ miR-87KO; miR-87; ttk69/+, ppk-Gal4, UAS-mCD8GFP/+; miR-87KO/ miR-87KO; ttk1e11/+; miR-87, hnf4/miR-87, ppk-Gal4, UAS-mCD8GFP/+; miR-87KO, hnf4Δ33/ miR-87KO; br/+; miR-87/miR-87, ppk-Gal4, UAS-mCD8GFP/brnpr-3; miR-87KO/ miR-87KO. (PDF) [file pgen.1008942.s003.pdf]

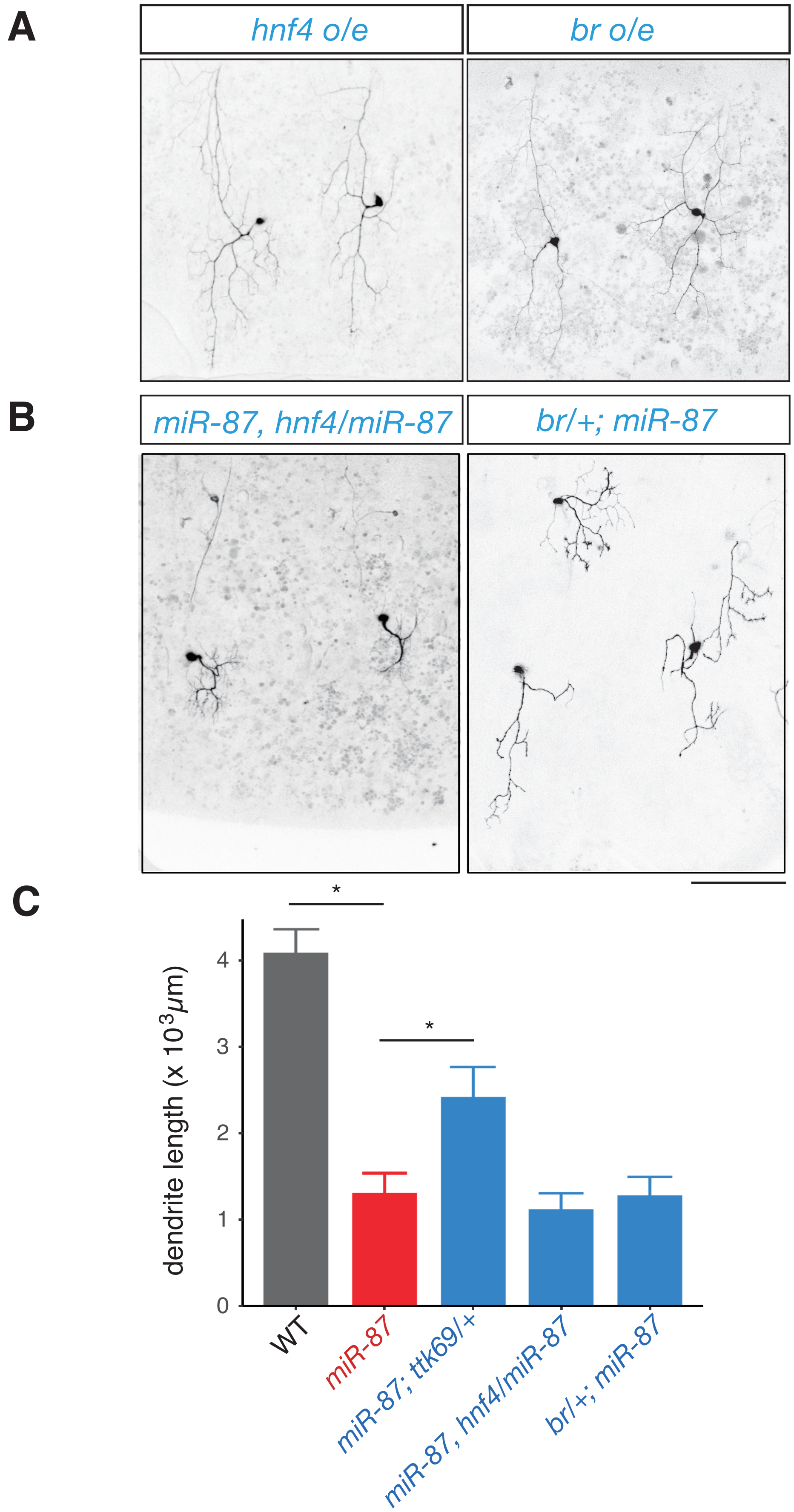

Supplemental Figure S3: *hnf4* and *br* are dispensable for dendrite regeneration defects in *miR-87* neurons.

(A) Overexpression of *hnf4* or *br* causes dendrite regeneration defects. Morphology of *hnf4* overexpressing (*hnf4* o/e) and *br* overexpressing (*br* o/e) dendrites at 72 hr APF. Scale bar = 100 μm. (B) Reduction of *hnf4* or *br* dosage causes no obvious rescue in dendrite regeneration defects in *miR-87* KO C4da neurons. Morphology of *miR-87* KO with reduction of one copy of *hnf4* (*miR-87*, *hnf4/miR-87*) or *br* (*br*/+; *miR-87/miR-87*) dendrites at 96 hrs APF. Scale bar = 100 μm. (C) Quantification of total dendrite length in control wild-type (WT), *miR-87* KO (*miR-87*), *miR-87* KO with reduction of one copy of *ttk69* (*miR-87*; *ttk69* /-), *miR-87* KO with reduction of one copy of hnf4 (*miR-87*, *hnf4/miR-87*), *miR-87* with reduction of one copy of *br* (*br*/+; *miR-87*) dendrites at 96 hrs APF. n = 15, Error bars indicate mean ± S.D., *p<0.01(ANOVA with a post hoc Bonferroni correction).

Kitatani et al. Supplemental Figure S3
