# Supplementary material for: Drosophila miR-87 promotes dendrite regeneration by targeting the transcriptional repressor Tramtrack69
Source: PLoS Genet. 2020 Aug 7;16(8):e1008942. doi: 10.1371/journal.pgen.1008942 (PMC7439810; doi:10.1371/journal.pgen.1008942)
Supplement: S4 Fig — (A) A schematic illustration of the ttk69 3’UTR sensor construct. (B) Regulated expression of ttk69 3’UTR sensor. Images show expression of the ttk69 3’UTR sensor in wild-type (WT) and miR-87 KO mutant (miR-87) C4da neurons at 2nd instar larval (2L) and white pupal (WP) additionally expressing ppk-CD4-tdTomato. Scale bar = 20 μm. (C) Quantification of the ttk69 3’UTR sensor GFP expression in C4da neurons at the indicated developmental stages. The ttk69 3’UTR sensor fluorescence intensity was normalized to the control sensor fluorescence intensity. n = 25, Error bars indicate mean ± S.D, *p<0.001 (Student’s t-test). Genotypes: ppk-CD4-tdTomato; miR-87KO/miR-87KO; tub-GFP-ttk69 3’UTR /+ (PDF) [file pgen.1008942.s004.pdf]

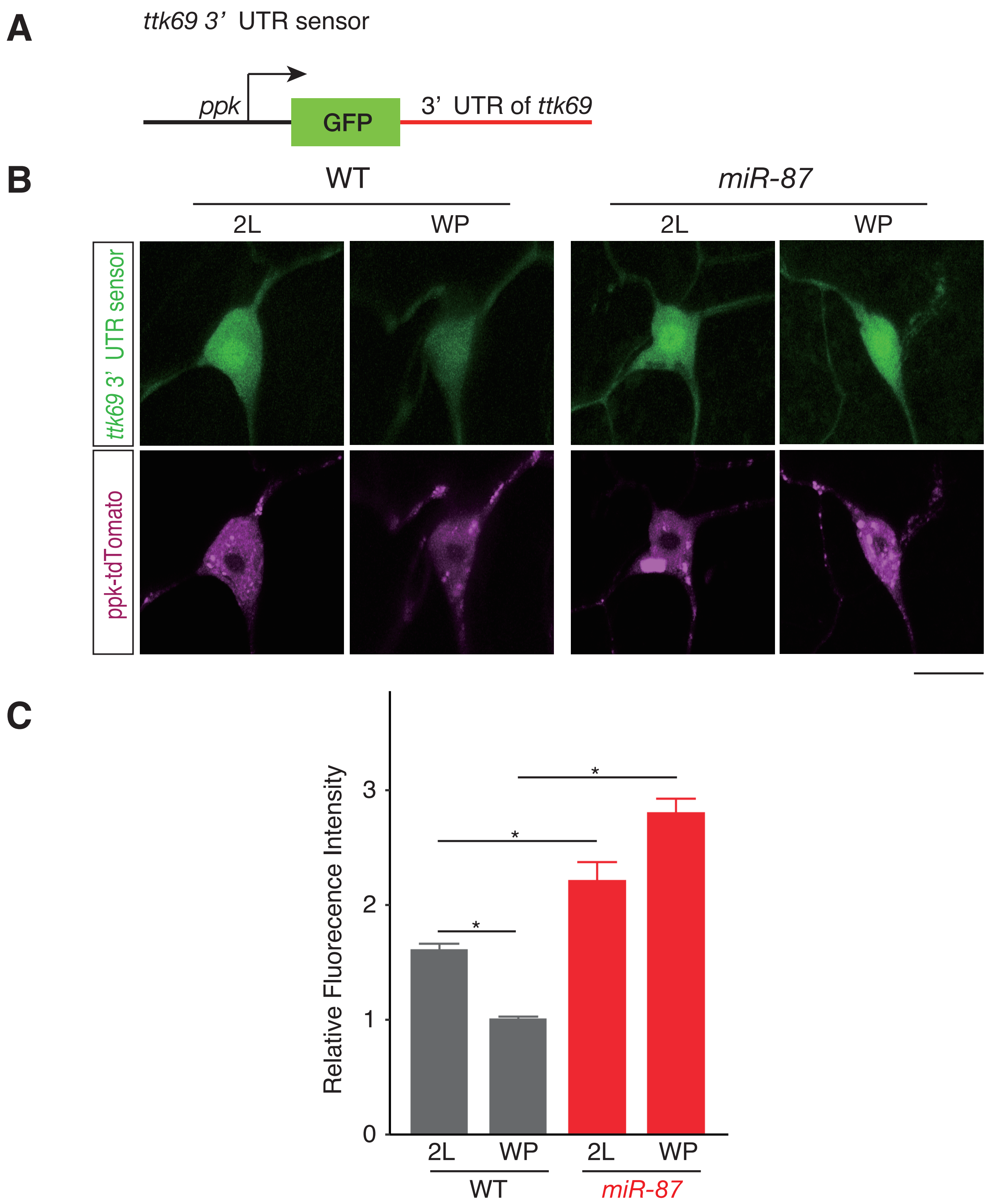

Supplemental Figure S4: Expression of *ttk69*-3' UTR sensor in C4da neurons is suppressed in the larvae-to-pupae transition

(A) A schematic illustration of the *ttk69*-3' UTR sensor construct. (B) Regulated expression of *ttk69* -3' UTR sensor. Images show expression of the *ttk69* -3' UTR sensor in wild-type (WT) and *miR-87* KO mutant (*miR-87*) C4da neurons at 2nd instar larval (2L) and white pupal (WP) additionally expressing ppk-CD4-tdTomato. Scale bar = 20 μm. (C) Quantification of *ttk69* -3' UTR sensor GFP expression in C4da neurons at the indicated developmental stages.The *ttk69* -3' UTR sensor fluorescence intensity was normalized to the control sensor fluorescence intensity. n = 25, Error bars indicate mean ± S.D, *p<0.01 (Student' s *t*-test).
