# Supplementary material for: Drosophila miR-87 promotes dendrite regeneration by targeting the transcriptional repressor Tramtrack69
Source: PLoS Genet. 2020 Aug 7;16(8):e1008942. doi: 10.1371/journal.pgen.1008942 (PMC7439810; doi:10.1371/journal.pgen.1008942)
Supplement: S5 Fig — (A, B) Overexpression of ttk69 causes dendrite regeneration defects in adult. Morphology of wild-type control (WT) and ttk69 overexpressing (ttk69 o/e) dendrites at adult 1 day (A). Scale bar = 100 μm. Quantification of total dendrite length in wild-type (WT), ttk69 overexpressing (ttk69 o/e) neurons (B). n = 15, Error bars indicate mean ± S.D, *p<0.01 (Student’s t-test). (C, D) Time-lapse images of dendrite regeneration after injury in wild-type (WT) and ttk69-overexpressed (ttk69 o/e) neurons. Red arrows indicate the site of dendrite severing in 2nd instar larvae (44–48 hr AEL) (C). Scale bar = 50 μm. Quantification of regenerated dendrite length after severing in control wild-type (WT) and ttk69-overexpressed (ttk69 o/e) neurons at 54 hr after severing. n = 6, Error bars indicate mean ± S.D., *p<0.05 (unpaired t-test). Genotypes: WT, ppk-Gal4, UAS-mCD8GFP; ttk69 o/e, ppk-GAL4, UAS-mCD8GFP; +/+; UAS-ttk69/+. (PDF) [file pgen.1008942.s005.pdf]

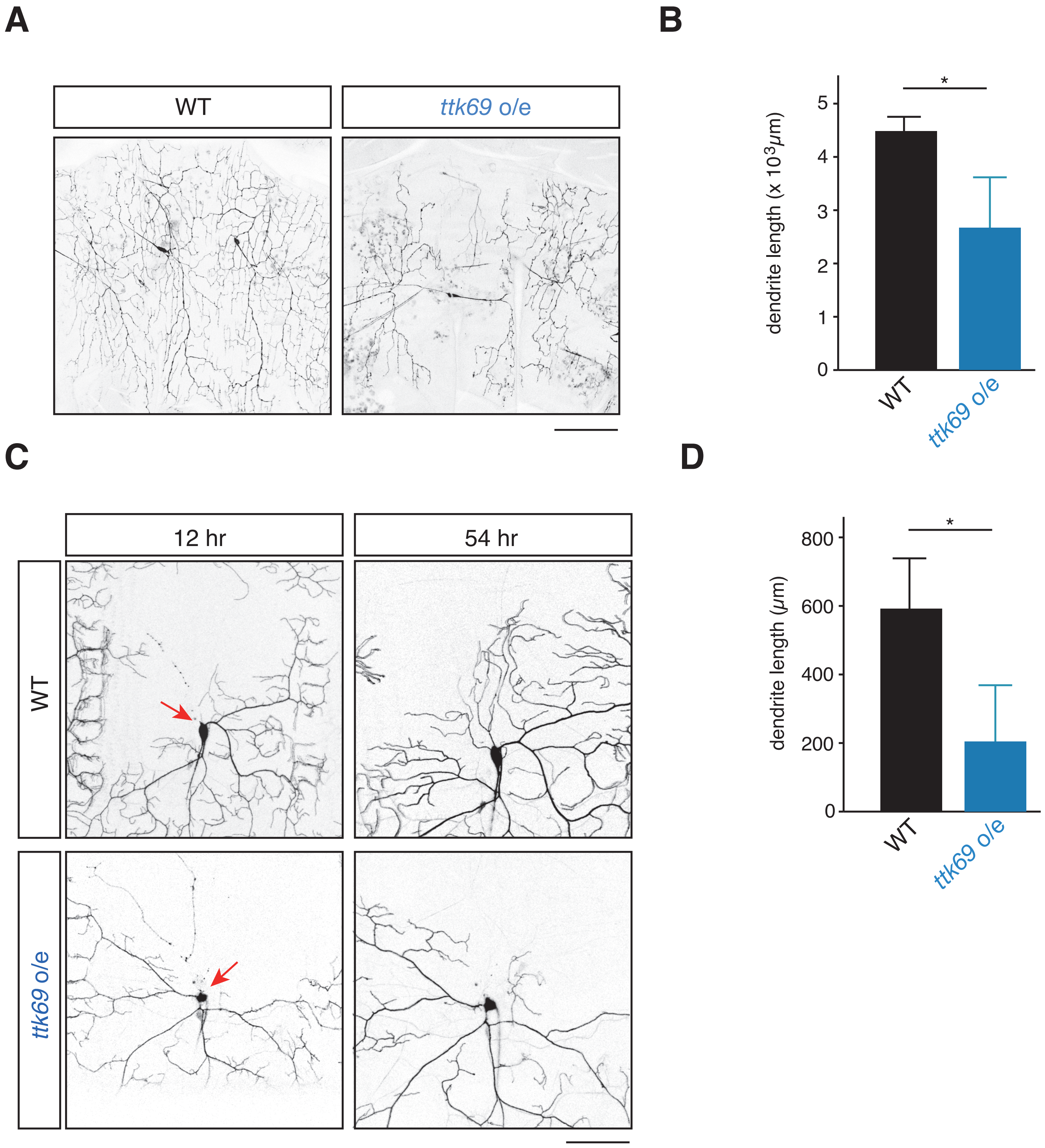

Supplemental Figure S5. *ttk69* overexpression causes dendrite regeneration defects after injury. (A, B) Overexpression of *ttk69* causes dendrite regeneration defects in adult. Morphology of wild-type control (WT) and *ttk69* overexpressing (*ttk69* o/e) dendrites at adult 1 day (A). Bar = 100 μm. Quantification of total dendrite length in wild-type (WT) and *ttk69* overexpressing (*ttk69* o/e) neurons (B). Error bar indicates means ± S.D., *p<0.01 (Student' s t-test). (C, D) Time-lapse images of dendrite regeneration after injury in wild-type (WT) and ttk69-overexpressed (ttk69 o/e) neurons. Red arrows indicate the site of dendrite severing in 2nd instar larvae (44-48 hr AEL) (C). Scale bar = 50 μm. Quantification of regenerated dendrite length after severing in control wild-type (WT) and ttk69-overexpressed (ttk69 o/e) neurons at 54 hr after severing. n = 6, Error bars indicate mean ± S.D., *p<0.05 (unpaired t-test).
