# Supplementary figures and images for: Drosophila miR-87 promotes dendrite regeneration by targeting the transcriptional repressor Tramtrack69
Source: PLoS Genet. 2020 Aug 7;16(8):e1008942. doi: 10.1371/journal.pgen.1008942 (PMC7439810; doi:10.1371/journal.pgen.1008942)

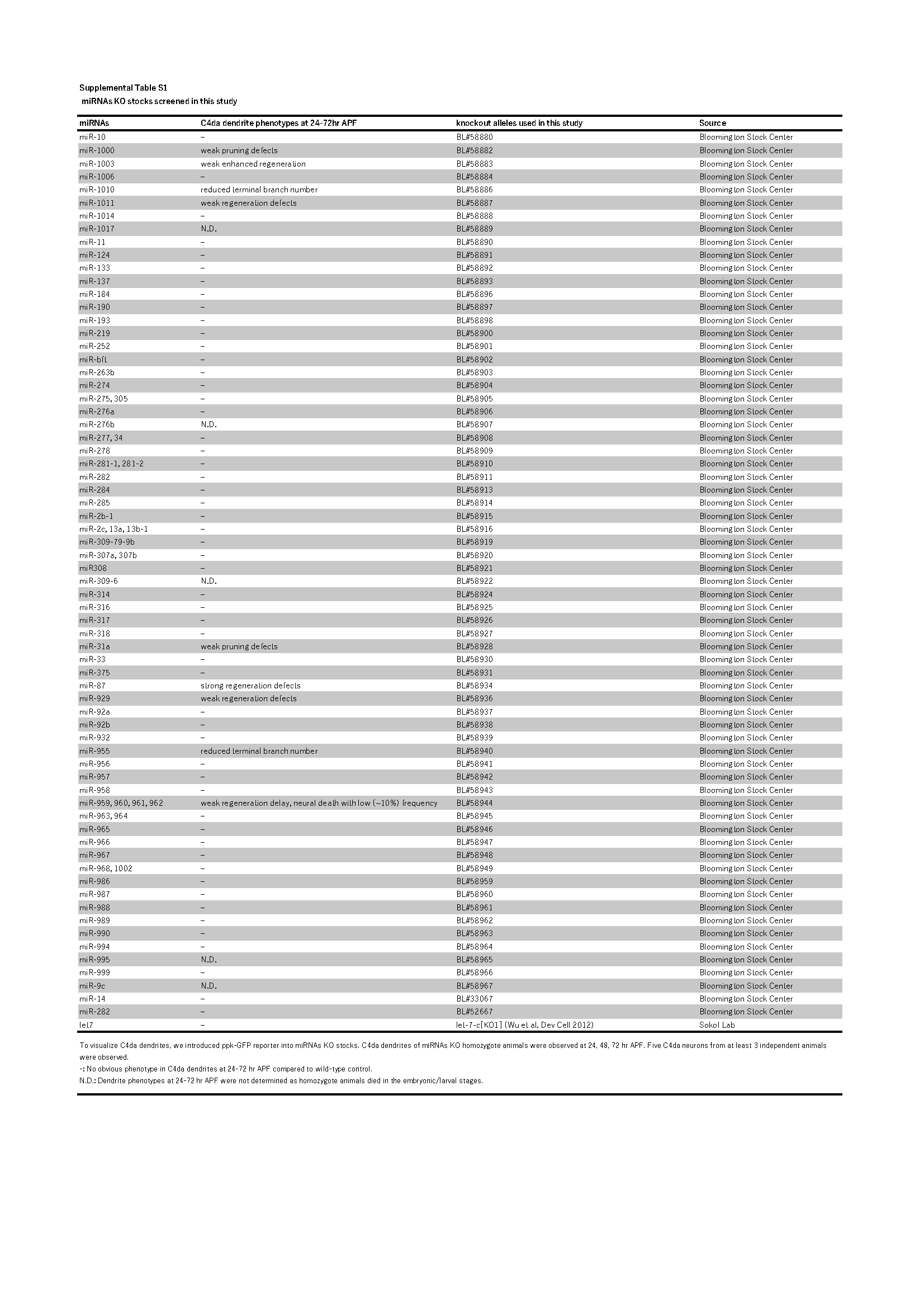

Supplement: S1 Table — To visualize C4da dendrites, we introduced ppk-GFP reporter into miRNAs KO stocks. C4da dendrites of miRNAs KO homozygote animals were observed at 24, 48, 72 hr APF. Five C4da neurons from at least 3 independent animals were observed. (TIFF) [file pgen.1008942.s006.tiff]

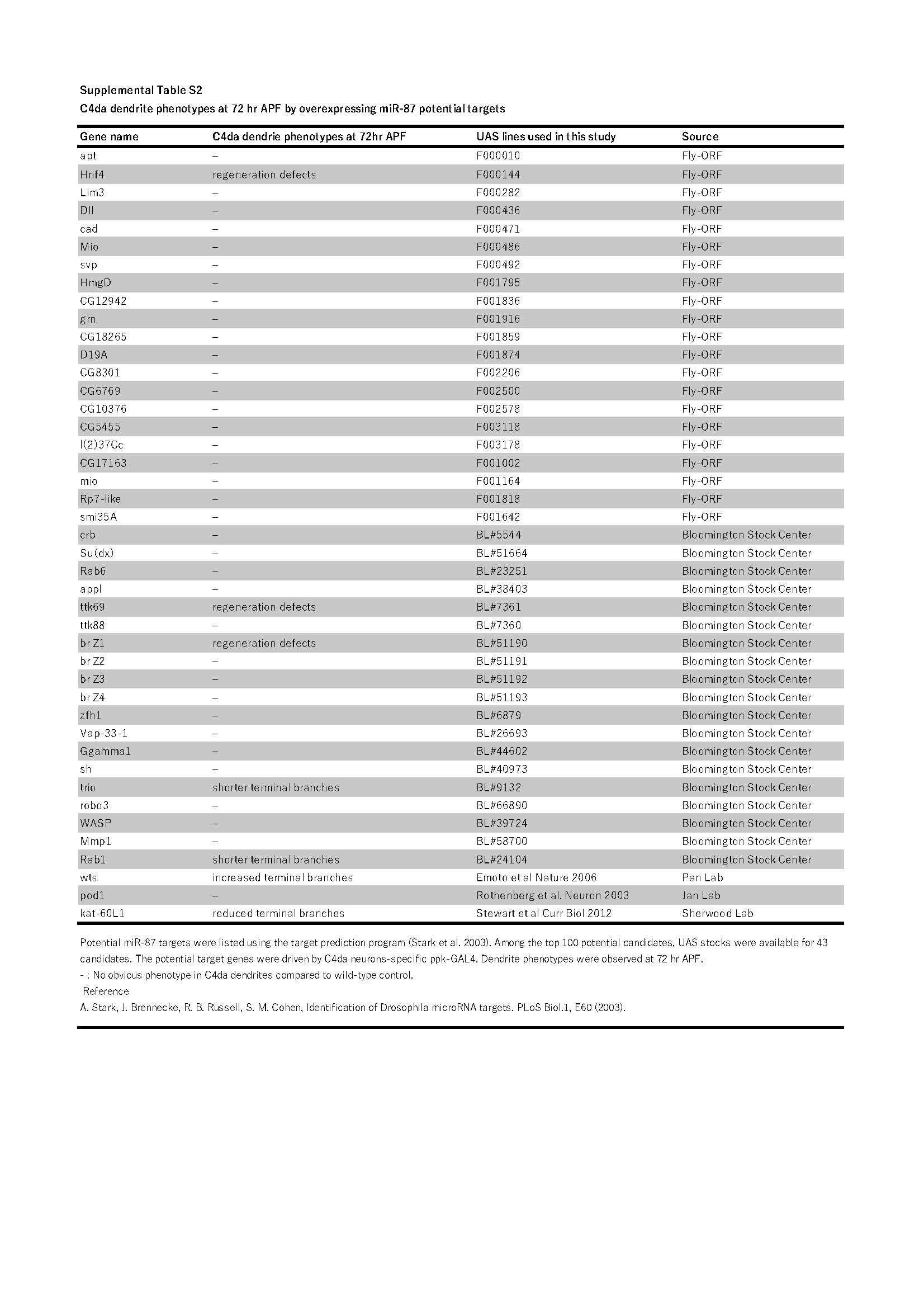

Supplement: S2 Table — Potential miR-87 targets were listed using the target prediction program (Stark et al. 2003). Among the top 100 potential candidates, UAS stocks were available for 43 candidates. The potential target genes were driven by the C4da neuron-specific ppk-GAL4. Dendrite phenotypes were observed at 72 hr APF. (TIFF) [file pgen.1008942.s007.tiff]
