# Supplementary material for: Drosophila miR-87 promotes dendrite regeneration by targeting the transcriptional repressor Tramtrack69
Source: PLoS Genet. 2020 Aug 7;16(8):e1008942. doi: 10.1371/journal.pgen.1008942 (PMC7439810; doi:10.1371/journal.pgen.1008942)
Supplement: Attachment — Submitted filename: 2020 Kitatani PLoS Genetics Rebuttal final submitted.docx [file pgen.1008942.s010.docx]

**Point-by-point responses to Reviewers**

We would like to thank the Editor and Reviewers for their positive and constructive comments on our paper. According to the Reviewers’ comments, we have added several new pieces of data and modified the text in ways that substantially improve the manuscript.

***Reviewer #1:*** *Summary: Kitatani, et al. present an interesting study showing that the microRNA, miR-87, is required for adult-specific dendritic growth during pupal metamorphosis. Loss of miR-87 impairs dendrite regeneration whereas over-expression of miR-87 leads to early initiation of regeneration. Moreover, miR-87 acts in a cell-autonomous manner to regulate dendritic growth. The authors present evidence consistent with Tramtrack 69 (ttk69) being a functional target of miR-87 and reducing ttk69 expression in miR-87 KO neurons restores dendritic growth. Finally, the authors show that miR-87 is required for dendrite regeneration after acute injury.*

***Major comments***
***1.)*** *Throughout the manuscript and figures the exact genotype of “wild-type” is not clear. This leads to uncertainty about whether proper controls were used for each experiment. Presumably the wild-type in Figures 1, 3 and 4 is a fly with the same genetic background as the miR-87 KO mutant. For Figures 5, 6, and 7 it is not clear if the wild-type control is a GAL4-only control, a UAS-only control, or some other type of genetic background control. Making the genotype for each control explicitly clear is essential.*

According to the review’s comments, we have added the detailed genotype of each fly line used in the legends of each figure.

***2.)*** *Figure 4 - The authors should examine fluorescence from the control GFP sensor in miR-87 KO flies during 2L and WP stages to see if there are uniform levels of GFP expression in C4da neurons. Further, the methods used by the authors to quantify fluorescence in Figure 4H are unclear. The authors should quantify fluorescence of the miR-87 GFP sensor in WT and miR-87 KO neurons and normalize the data to fluorescence intensity of the control GFP sensor lacking miR-87 binding sites. This would demonstrate additional confidence that control of the miR-87 GFP sensor is dependent on miR-87 activity.*

According to the Reviewer’s comments, we normalized the expression of the *miR-87* sensor to expression of the control sensor in both WT and *miR-87* KO neurons. We have added the data and details on our method for quantification in Figure 4H and the accompanying legend.

***3.)*** *Figure 6 - The authors indicate that over-expression of three miR-87 targets, ttk69, hnf4, and br, caused significant defects in dendrite regeneration. They follow up with dosage-sensitive interactions between the candidate genes and miR-87. However, only data for ttk69 is provided. Similar data for hnf and br should be shown. Specifically, assaying whether heterozygous loss-of-function mutations in hnf and br could suppress the regeneration defect in miR-87 KO neurons. If all three candidate genes show similar levels of suppression, the manuscript should specifically address why ttk69 was chosen for follow-up experiments. If the loss-of-function studies were inconclusive, those data should be included.*

According to the Reviewer’s comments, we have added the representative images depicting C4da dendrite arbors in flies overexpressing *hnf4* or *br* in C4da neurons in Supplemental Figure S3A. We have also added genetic interaction data demonstrating that, unlike *ttk69*, reduction of a copy number of *hnf4* or *br* in the *miR-87* KO background caused no obvious changes in the *miR-87* dendrite regeneration phenotypes. We added these data in Supplemental Figures S3B and S3C and included a brief discussion of these results in our revised text (Lines 252-254).

***4)*** *The supplemental videos are helpful, but I was struck by how the dendrites in the mutants appeared to fasciculate more than the wild-type. Given that these dendrites typically exhibit self-avoidance, in order to tile in the animal, could the authors comment on whether that is an observation that was made in the other live imaging studies? Is there a way to measure number of contacts observed? I do not need the authors to add additional data, commenting on this possibility would be sufficient, but if they have that data tabulated, adding it would be good.*

According to the Reviewer’s comments, we have checked multiple movies of wild-type and *miR-87* dendrite regeneration and found no obvious dendrite retraction was observed in *miR-87* dendrites. Rather, they seemed to be stuck to elongate thick regions. It is thus likely that the dendrite regeneration defects in *miR-87* is unrelated to the contact-dependent retractions. We have mentioned this in our revised Results section (Lines 182-185).

***5)*** *The authors provide compelling evidence that ttk69 interacts genetically, and evidence that levels of ttk69 are elevated in miR-87 mutants. A complete proof that miR-87 regulates ttk69 post-transcriptionally would likely necessitate manipulating the 3’UTR binding sites in ttk69 and rescuing that by mutating miR-87 to recover binding and regulation. Thus, I would ask the authors to simply mention in the discussion that their evidence is consistent with the 3’ UTR regulation, but that there are potentially other factors that miR-87 could be regulating upstream of ttk69.*

According to the comments, we monitored effects of *miR-87* on expression of a *ttk69*-3’UTR (Huang et al. Development 2013). We found that, similar to the *ttk69* qPCR data (Figure 6E), the GFP intensity of the *ttk69*-3’UTR sensor was significantly suppressed at WP compared to 2L, and this suppression was undetectable in *miR-87* KO background. Hence, ttk69 is likely regulated post-transcriptionally via a mechanism that involves *miR-87* control of the *ttk69* 3’UTR. We had added these data in Supplemental Figure S4A-4C, and presented these new data in the revised Results (Lines 264-277).

***Minor comments***
***1.)*** *Figure 1- Ideally, the authors should show representative images for dendrites at 48-hr APF and in 2-day and 3-day-old adults. Perhaps it would be easier to break this figure up into two figures, one for dendritic growth after puparium formation and a second figure for dendritic growth post-eclosion.*

We added representative images of 2- and 3-day old wild type and *miR-87* mutant C4da neurons in Supplemental Figure S1.

***2.)*** *p.8, 2nd paragraph - AFP should be APF*

We corrected this error.

***3.)*** *p.10, 1st paragraph. Figure call-out error “…by the miR-87 sensor beginning in 3rd instar larvae, resulting in a >50% reduction of GFP expression in early pupal stages (Fig 4B-4G). However, 4B and 4C have control sensor data.*

We have corrected the figure callout to Figure 4D-4H.

***4.)*** *p.15, 2nd paragraph - “…suggesting that the increased expression of miR-87…”. Expression of miR-87 was never demonstrated, only increased activity through the GFP sensor.*

We have changed “increased expression” to “increased activity”.

***5.)*** *In Figures 5, 6, and 7 the genotypes of each fly line are not clear. It would be helpful to write out the genotypes in the figure legend similar to the genotypes of MARCM clones in Figure 2. “Clone genotypes: (WT) hsFLP, ppk-Gal4, UASmCD8GFP/+; FRT40A, (miR-87) hsFLP, ppk-Gal4, UAS-mCD8GFP/+; miR-87, FRT40A, and (miR-87 + UAS-miR-87) hsFLP, ppk-Gal4, UAS-mCD8GFP/+; miR-87, FRT40A; UAS-miR-87/+.”*

We updated all of the figure legends to included details of experimental genotypes.

***5.)*** *A section in the Methods should be added for quantification of GFP fluorescence in Figures 4 and 7.*

We have added the information in the legends for Figures 4 and 7.

**6.)** The authors quantify dendritic field size in Figure 2, but not in any other experiments. I did not see a rationale for this.

We monitored dendritic field size in addition to dendrite length as an additional criteria to evaluate dendritic phenotypes in the single cell MARCM clones; monitoring field size in other contexts (ie, when all C4da neurons are labeled) is somewhat confounded by the presence of intermingling branches from neighboring segments at segment boundaries. We thus would like to keep these data in Figure 2.

***7.)*** *p.30, Figure 3 legend - “Time stumps” should be “Time stamps”*

We have corrected this typographical error.

***8.)*** *Could the authors please comment on when the pickpocket enhancer becomes active in the C4da neurons?*

The *ppk* enhancer becomes active in C4da neurons during late embryonic stages. We have added the information to the revised text (Lines 128-129).

***9.)*** *Could the authors please confirm that the total dendritic length is the sum of all the dendrites from a single neuron. Or is it from multiple neurons within the same animal?*

Total dendritic length reflects the sum of branch lengths from all branches of a single neuron. We have added this information in the figure legend.

***Reviewer #2:*** *This study has demonstrated that microRNA miR-87 contributes to neuronal dendrite outgrowth in both developmental and injury-induced regeneration and has shown its underlying molecular mechanism at least at the level of identifying a critical target of miR-87, ttk69. All of the experiments were done in scientifically sound manners and the data are appropriately presented.*

***Minor comments:
1.)*** *What is the role of the miR-87 target, ttk69, in dendrite morphogenesis and regeneration of C4da neurons? The authors state roles of ttk69 in C1da neurons and R7 photoreceptor. If the role of ttk69 in C4da neurons is not known, state as such.*

We are not aware of any reports of Ttk69’s role in dendrite morphogenesis or regeneration of C4da neurons (although prior reports have investigated functions of ttk69 in other neurons), and we have updated our discussion to reflect this fact (Lines 355-356).

**2.)** *The positive hits of the primary screening of the miR-67 targets were hnf4, br and ttk69 (Figure 6A and 6B) and the authors examined ttk69 in their secondary test (Figure 6C and 6D). What happened to hnf4 and br? If they were negative in the secondary test or if the authors did not examine those candidates further, state as such.*

We examined whether reduced copy number of *hnf4* or *br* might affect dendrite regeneration defects in *miR-87* KO mutants and found no significant changes in dendrite regeneration. We added these new data in Supplemental Figure S3B and S3C.

**3.)** *It is preferable to describe the clone genotype in Figure 5 legend (did I overlook it?)*

The information was missing from the initial submission, but we have added experimental genotypes to each figure legend.

***Reviewer #3:*** *Drosophila miR-87 promotes dendrite regeneration by targeting the transcriptional repressor Tramtrack69 (PGENETICS-D-20-00327)

In this manuscript the authors provide genetic evidence to report that microRNA miR-87 is required not only for the developmentally programed dendrite regeneration of Drosophila sensory neurons during metamorphosis, but also for the injury-induced dendrite regeneration of larval neurons. By monitoring the miR-87 sensor, they further show that the activity of miR-87 is upregulated in neurons during larvae-to-pupae transition, prior to dendrite regeneration, and also in larval neurons after dendrite injury. Finally, they identify one of the miR-87 targets, ttk69, and show that miR-87-mediated reduction of ttk69 is required to promote both the developmentally programed and the injury-induced dendrite regeneration.

The genetic studies of this manuscript are well designed and carried out. The miR-87 activity reported by the miR-87 sensor is nicely shown correlated with the ability of dendrite regeneration in C4da neurons, and with the reduction of ttk69 expression in neurons. The manuscript would need some improvement for it to be accepted by the journal. Here are the two main issues that I would like to raise for this manuscript.*

*First, the authors have done a lot for the developmentally programed dendrite regeneration; however, less has been done for the injury-induced dendrite regeneration of larval neurons. To support their main conclusion that a common intrinsic mechanism, ttk69 downregulation by miR-87 to promote dendrite growth in C4da neurons, is shared by both cases, the authors need to provide more evidence for the dendrite regeneration in larval neurons after injury.*

***1.)*** *In Fig 5, the authors show that miR-87 overexpression could cause precocious dendrite growth in C4da neurons at 30 hr APF. Does miR-87 overexpression also cause precocious dendrite growth in larval C4da neurons after injury?*

According to the comments, we examined these points and found that *miR-87* overexpression in wild-type C4da neurons tended to enhance dendrite regeneration after injury, although the difference between the group means did not reach the threshold for “significance” of p<0.05). We have added these data to our revised manuscript as Figure 7B and discussed them in our revised Results section (Lines 294-297).

***2.)*** *In Fig 6, the authors show that ttk69 overexpression causes dendrite regeneration defects in C4da neurons at 72 hr APF. Does ttk69 overexpression also cause impaired dendrite growth in adult C4da neurons, like the ones in miR-87 KO adults (Fig 1)? Does ttk69 overexpression also cause impaired dendrite growth in larval C4da neurons after injury?*

Overexpression of *ttk69* in C4da neurons caused a reduced dendrite length in 1-day adults as well as at 72 hr APF. We added these data to our revised submission as Supplemental Figure S5A and S5B. We also induced injury in *ttk69*-overexpressing C4da neurons and found that dendrite regeneration after injury was significantly impaired. These data are added in Supplemental Figure S5C and S5D, and mentioned in our revised Results section (Lines 297-301).

*Second, the current results provided in this manuscript only suggest that ttk69 is likely one of the miR-87 target genes for dendrite regeneration. Although the authors show in Fig 6 that no decrease of ttk69 mRNA in the brain extracts of miR-87 KO animals at white pupal stages, this result still indirectly suggest that ttk69 is one miR-87 target in the brain. If the authors could provide evidence to demonstrate that ttk69 is the real target of miR-87 in C4da neurons, it would greatly strengthen the mechanistic insights into this manuscript.*

According to the comments, we monitored expression levels of a *ttk69*-3’UTR sensor in which the *ttk69*-3’UTR sequence is fused with GFP at 3’UTR region (Huang et al. Development 2013). We found that, similar to our *ttk69* qPCR results (Figure 6E), the GFP intensity of the *ttk69*-3’UTR sensor was significantly suppressed at WP compared to 2L, and this suppression was undetectable in the *miR-87* KO background. We had added these data in Supplemental Figure S4B and S4C and presented them as a new paragraph in our revised Results (Lines 264-277).

***Other points:***
***1.)*** *Do the authors try to rescue dendrite regeneration defects in miR-87 KO neurons with removal of one copy of hnf4 or br, or with RNAi of either genes? Or any combination of these three candidate genes reduction to see whether it can improve the rescue efficiency of dendrite regeneration defects in miR-87 KO neurons by single gene reduction?*

As indicated above, we assayed whether reduction of copy number of *hnf4* or *br* might affect dendrite regeneration in *miR-87* KO neurons and found no significant changes in dendrite regeneration. We added these data in Supplemental Figure S3B and S3C.

***2.)*** *On page 9, the cited information in the sentence, “A previous northern blot analysis has shown that miR-87 expression in the brain ……”, is not quite correct, according to ref 40. They used RNA from the whole animals, not the brain, at various developmental stages for northern blot analysis.*

We changed this statement from “in the brain” to “in whole animals” to accurately reflect the information from the citation.

***3.)*** *In the Discussion, the authors discuss the possible mechanism of temporal regulation of miR-87 expression dynamics, and think ecdysone might control the temporal expression of miR-87. However, according to the information in ref 40, the experimental manipulation of ecdysone or Juvenile hormone signaling in S2 cells (done by the authors of ref 40) does not affect the expression of miR-87. I think the authors should include this information and discuss other possibility that could regulate miR-87 expression temporally.*

We thank the reviewer for giving us the opportunity to clarify our model. We hypothesize that miR-87 expression is linked to the stage-specific ecdysone-responsive transcription factors that are induced in prepupae. To clarify this point, we added the following text to our discussion:

Studies in S2 cells suggest that a direct connection between ecdysone and miR-87 is unlikely (40). Instead, ecdysone-responsive genes that are absent in S2 cells likely induce miR-87 expression to trigger dendrite regrowth. Indeed, temporal specificity of ecdysone signaling is imparted by stage-specific induction of ecdysone-responsive transcription factors including Eip75B, HR3, and HR4, each of which are induced by the late larval ecdysone pulse (55) and absent or lowly expressed in S2 cells (56). (Lines 383-390)

***4.)*** *The results shown in Fig 2 demonstrated that dendrite regeneration defects were largely, not fully, rescued by UAS-miR-87 expression in the miR-87 MARCM clones of C4da neurons, suggesting that mir-87 has a role acting cell-autonomously in neurons for dendrite regeneration. Since it is not fully rescued by UAS-miR-87 expression in miR-87 MARCM clones, compared to the wild-type clones, it may suggest a possibility that miR-87 might also have a cell-non-autonomous role in dendrite regeneration. Have the authors done any experiments to rule out this possibility? Or can the authors discuss it in the Discussion?*

Although our results in this figure and in figure 7B suggest that resupplying *miR-87* expression to neurons largely restores dendrite regrowth to control levels, we cannot rule out the possibility that cell non-autonomous functions of miR-87 contribute to dendrite regrowth. We have reworded our discussion of the MARCM rescue results to reflect this fact (Lines 155-159).
